# Attitudes and barriers to pelvic floor muscle exercises of women with stress urinary incontinence

**DOI:** 10.1186/s12905-022-02067-4

**Published:** 2022-11-26

**Authors:** Wilai Sawettikamporn, Sirirat Sarit-apirak, Jittima Manonai

**Affiliations:** 1 Ramathibodi Hospital, Bangkok, Thailand; 2 Department of Obstetrics & Gynaecology, Faculty of Medicine Ramathibodi Hospital, Mahidol University, 270 Rama VI Road, Ratchathewi, Bangkok, 10400 Thailand

**Keywords:** Pelvic floor muscle exercise, Stress urinary incontinence, Attitude, Barrier, Motivator, Qualitative research

## Abstract

**Background:**

Pelvic floor muscle exercise (PFME) is a first-line treatment for stress urinary incontinence (SUI), but adherence to PFME is often problematic. The aim of this study was to better understand the attitudes and barriers to practicing pelvic floor muscle exercise among women with SUI.

**Methods:**

We conducted a qualitative study using semi-structured interviews. Purposive sampling was used to approach eligible participants. The interview included questions focused on women’s perceptions regarding SUI and PFME, sources of information, support, and barriers and motivators of PFME. In-depth interviews were conducted until data saturation occurred. After several readings of written interview transcripts, codes were retrieved, and thematic analysis was conducted.

**Results:**

Seven women participated in the study (average age 53.2 years), and most (4/7) were retired. Three salient themes emerged from the data: (1) perception of SUI, (2) barriers to PFME, and (3) motivators to exercise. Participants highlighted various barriers to PFME: (1) lack of self-discipline owing to both intrinsic and extrinsic factors, (2) lack of confidence in how to perform the exercises properly, and (3) skepticism regarding the efficacy of treatment according to women’s direct and indirect experiences. Achievement of desired outcomes, symptom severity, women’s expectations, and fear of surgery were motivators to regularly perform PFME.

**Conclusions:**

The main barriers to regular PFME were inadequate self-discipline, knowledge, and confidence in performing the exercises, and a poor perception about the effectiveness of PFME.

**Supplementary Information:**

The online version contains supplementary material available at 10.1186/s12905-022-02067-4.

## Background

Stress urinary incontinence (SUI) is the most common urinary complaint among women and is defined as an unintentional loss of urine on effort or physical activity, such as sneezing or coughing [1]. Pelvic floor muscle training (PFMT), which aims to maximize the functional capabilities of pelvic floor muscle (PFM) is an effective first-line treatment for SUI [2]. Women with SUI who regularly practice pelvic floor muscle exercises (PFME) are eight times more likely to have no SUI symptoms compared with no treatment or inactive control treatments [3]. Exercise adherence is crucial to improving PFM function and has been identified as a main predictor of its long-term effectiveness [3, 4]. However, high adherence to treatment or adhering exactly to instructions is multifaceted and requires active patient participation and cooperation. Moreover, health behavior is influenced by family members, healthcare providers, the health system, socio-economic context, and cultural background [5]. Although theories and strategies for promoting adherence have been widely studied [6–8], adherence to PFME protocols is often problematic [9, 10]. Clinical trials have assessed the efficacy of different strategies to enhance PFME adherence including educational techniques, mobile applications and vaginal devices; however, regarding adherence and incontinence symptoms have not been promising [11–14].

Regarding strategies to assist the uptake of PFME among women with SUI, clinicians’ perspectives have been studied and suggestions for effective approaches proposed [15–17]. However, understanding patients’ perspectives would guide appropriate behavioral change interventions to improve PFME adherence, resulting in increased treatment effectiveness and better patient quality of life. Previous studies investigated patients’ experiences and determinants of adherence have mainly been conducted among postnatal women as well as middle-aged and older women from Western countries [9, 17, 18]. There is insufficient evidence regarding those factors that have an impact on adherence to a home-based exercise program for SUI, with a culturally diverse perspective. Therefore, it is important to increase our understanding of the perspective Asian women regarding PFME in terms of knowledge, skills, capability, and motivation. The aim of this qualitative study was to better understand the attitudes and barriers to PFME among Thai women with SUI.

## Methods

### Study design and participants

We conducted a qualitative descriptive study using semi-structured interviews administered at a urogynecology clinic of a university hospital in Thailand. Participants were patients with predominant SUI attending the clinic during the study period. The diagnosis was confirmed by attending physicians determined by history taking, physical examination and 3-day bladder diary. The severity of incontinence was assessed using a four-level severity index [19]. Additionally, pelvic organ prolapse was assessed using the Pelvic Organ Prolapse Quantification (POP–Q) System [20].

Initially, women with urinary incontinence were individually trained in the clinic by the urogynecology nurse specialist (SS). Vaginal palpation was used to assess the PFM function and muscle strength. The PFMT program included one (8–12 high intensity contractions or 5–10 min long) or more sets of exercises per day at home, performed on at least several days of the week, for at least 12 weeks. The leaflet for PFMT practice included the anatomy and function of PFM, written instructions for contracting PFM, and adherence recording was also distributed.

Purposive (nonprobability) sampling was used to select participants who were age 18 and older, diagnosed with SUI and had completed 12 weeks of PFMT session, and willing to be interviewed. Patients with predominant urgency urinary incontinence, advanced stage pelvic organ prolapse or a history of incontinence surgery were excluded. Eligible patients were approached by researchers and provided with a participant information sheet. Those agreeing to participate gave their written informed consent prior to the interviews. Then, a date and time for the interview was scheduled. The sample size was complete when data saturation occurred, i.e., when no new information could be obtained [21].

### Data collection

Semi-structured interviews were conducted using a face-to-face format and information about participants’ characteristics were collected. After reviewing the research’s objective and related literature, the scope of the interview and specific questions were determined. Then, a stepwise approach to developing an interview guide framework from research objectives was followed [22]. The contents of the question list were approved by an experienced, female urogynecologist (JM). Afterwards, the final interview guide was developed and tested in two female volunteers. After pilot testing, no new topics emerged, and the interview guide was demonstrated to effectively address the research objective (Additional file 1). The final interview guide included questions focused on women’s perception regarding SUI and PFME, source of information, support, and barriers and facilitators of PFME.

The interviews were conducted by the principal investigator (WS) who is a female, registered nurse and was formally trained in in-depth interviewing. Participants had not met or received nursing care or education from the interviewer. The interviews took approximately 30 min and were conducted in a in a quiet, private room in the hospital to ensure a comfortable interview environment [23]. During the interviews, the collected data were audio recorded and field notes were also taken. Later, the interviews were transcribed using true verbatim transcription. Repeat interviews were undertaken if the information was insufficient or unclear. Participant or informant feedback on the findings was provided to establish study credibility. Two reflexive exercises were carried out among three female investigators, who were asked to state their perspectives; in this way, we could identify any issues that might affect the interview and data analysis processes [24].

### Data analysis

After several readings of the written interview transcript, codes were retrieved by two researchers (SS and JM). Significant codes were identified through line-by-line highlighting using colored ink pens. Then, thematic analysis following Braun and Clarke was conducted [25]. Once all transcripts had been coded, the researchers read and re‐read the coded content and organized data into meaningful groups according to the emerging key findings. Comparisons between each coder interpretations were conducted to understand similarities and differences, at that point discussions on areas of disagreement were made in order to validate the findings. The different codes were sorted into potential themes, and an initial thematic map was created. Next, all researchers reviewed, discussed and refined the coding framework using the transcripts and field notes. An inductive approach was used to identify themes without attempting to fit the codes into pre-existing analytic preconceptions. Finally, the themes and sub-themes were defined and named to improve clarity for the reader. To preserve participants’ anonymity, quotations were identified using a participant number.

### Ethical approval

The Institutional Review Board, Faculty of Medicine Ramathibodi Hospital approved the study (ID 02-61-84 MURA2018/169). Before starting the interview, each participant was briefed on the voluntary nature and purpose of the study. Information on how the interview data would be used, who would have access to the data, and whom participants could contact with questions was included in the informed consent documents.

## Results

Between June and October 2019, 12 patients with SUI were invited to participate, and 2 declined due to their inconvenience. All participants were classified as having moderate or severe SUI, according to the Sandvik index. After interviewing seven participants, no new information could be obtained, which indicated data saturation. The average participant age was 53.2 years and most (4/7) women were retired (Table 1). All women were multiparous and had at least a college degrees. A leaflet on PFMT had been given to all participants and all seven women had participated in PFMT supervised by a urogynecologist or a continence nurse.

**Table 1:** Participants’ sociodemographic profile

Pseudonym	Age range (years)	Marital status	Para	Occupation	Education level	SUI severity* (score)	Pelvic organ prolapse stage
ID 1	60’s–70’s	Married	2	Retired	Bachelor's degree	4	II
ID 2	60’s–70’s	Married	2	Retired	Associate’s Degree	3	II
ID 3	40’s–50’s	Married	1	Teacher	Bachelor's degree	8	I
ID 4	40’s-50’s	Married	2	Employee	Master's degree	4	I
ID 5	60’s–70’s	Widowed	3	Retired	Bachelor's degree	6	I
ID 6	60’s–70’s	Married	3	Retired	Bachelor's degree	7	II
ID 7	40’s–50’s	Married	2	Government officer	Master's degree	6	I

*3–6: moderate incontinence

8 or 9: severe incontinence

All interviews lasted about 20 and 30 min. Repeat interviews were conducted with two participants for clarification and to obtain additional information. Three salient themes emerged from the data: (1) perceptions of SUI (2) barriers to PFME, and (3) exercise motivators.

### Perceptions of stress urinary incontinence (SUI)

**Fig. 1:**
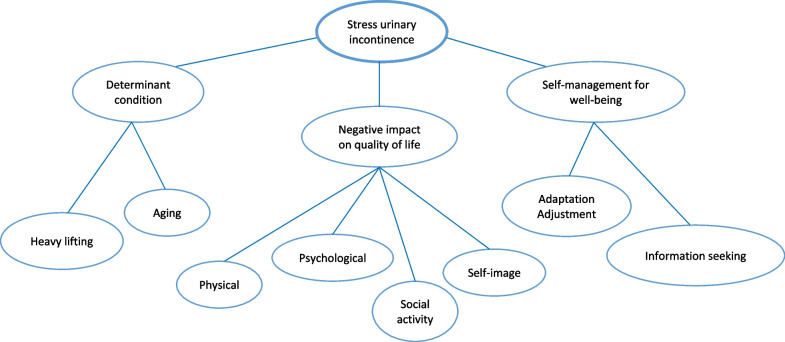
Patients’ perception of stress urinary incontinence

All participants had received information from their physicians and nurses regarding diagnosis and treatment alternatives. They stated that their incontinence symptoms had a negative impact on their quality of life and made them feel embarrassed and upset. Three sub-themes emerged: risks factors for SUI, impact on quality of life, and self-management (Fig. 1).

#### Subtheme 1: risk factors that are both preventable and non-preventable

In terms of participants’ knowledge, they were aware of their risk factors for developing SUI, which were heavy lifting and aging.

*“I know that the reason is because I have been carrying my grandchildren.” *(ID 1)

*“At first there was no problem… but after going to the farm, lifting heavy things…”* (ID 2)

*“I’m old…… my body is aging… it is sagging.”* (ID 4)

#### Subtheme 2: negative impact on quality of life

In general, participants considered that SUI negatively affected their physical and psychological health, as well as social activities.

*“It is very distressing when coughing or sneezing.”* (ID 2)

*“I feel insecure…..feeling anxious…….and then there may be a mess.”* (ID 6).

Women’s self-image and self-esteem were also diminished accordingly.

“….it must be smelly…it is disgusting for people around me.” (ID 7)

#### Subtheme 3: Self-management

Some participants tried to adjust their daily tasks so as to live with their undesirable symptoms. Some performed activities aimed at managing their condition and applied the skills necessary to maintain adequate psychosocial functioning.

*“If going outside……must carry pads or sanitary napkins.”* (ID 1)

*“If I have to cough, …quick…look for a chair to sit on or stand with my legs crossed.”* (ID 4)

Generally, participants with urinary incontinence did not seek help from others. Some sought condition-related information from the Internet, friends and relatives.

*“….ask my daughter to find out if there is any hospital or clinic that can help.”* (ID 2)

*“….talk to friends……why do I leak? Is there anybody else?”* (ID 6)

### Barriers to pelvic floor muscle exercise (PFME)

**Fig. 2:**
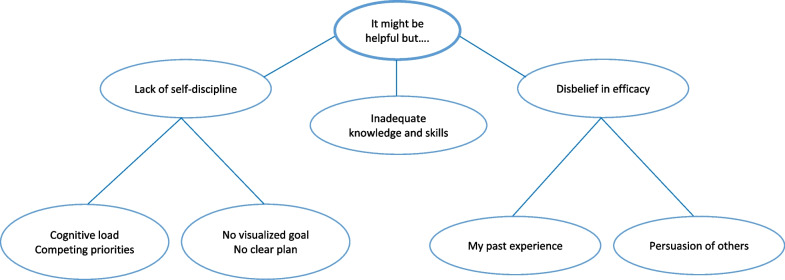
Barriers to pelvic floor muscle exercise

Participants discussed a number of factors affecting their adherence and highlighted various barriers to practicing PFME. These are summarized in Fig. 2.

First, the women admitted a lack of self-discipline owing to competing priorities in their busy schedules (extrinsic factor) and lack of goal visualization (intrinsic factor). Consequently, they forgot to do their exercises. Once their PFME routine was interrupted, it became difficult to make exercising a more consistent part of their routine again.

*“….sometimes we are busy… work hard…."* (ID 6)

*“.....can’t see my goal…why should I do...."* (ID 3)

Second, some participants did not know how to perform PFME properly. They could not recall their providers’ advice or feedback regarding pelvic floor muscle contraction or relaxation during the training sessions.

*“I don't know how to do. How can I tighten it?"* (ID 5)

*“I don't know which part to draw in.”* (ID 2)

Lastly, participants did not believe in the efficacy of treatment according to their own past experience when they practice PFME on their own, and indirect experience or opinions of other people or from books.

*“I used to exercise frequently but my problem didn't disappear.”* (ID 2)

*“I think it is impossible.”* (ID 7)

### Exercise motivators

**Fig. 3:**
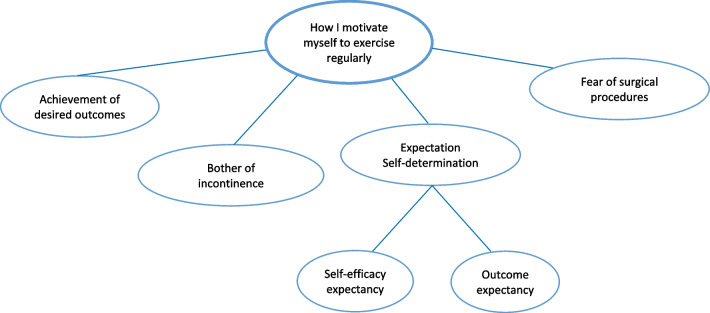
Pelvic floor muscle exercise motivators

In general, women with SUI disclosed that their motivation was highest after receiving guidance from healthcare providers but this gradually decreased afterwards. Participants stated that self-perceived efficacy, severity of symptom, expectation, and fear of surgery were motivators to regularly perform PFME (Fig. 3).

#### Subtheme 1: achievement of desired outcomes

When participants reached their goal of treatment or experienced symptom improvement, they were encouraged to adhere to their PFME schedule. They felt that quick success or rapid improvement gave them hope for long-term success.

*“….it gets better.”* (ID 1)

*“I feel……muscles become stronger.”* (ID 2)

#### Subtheme 2: symptom severity

Participants who experienced more severe incontinence symptoms tended to prioritize adherence to PFME and keep it at on the top of their list of daily activities. This information revealed the influence of symptom severity on long-term adherence.

*“….even a single cough… I can’t hold it.”* (ID 2)

*“…..It seems like there is dampness on every chair in every seat I sit on.”* (ID 7)

#### Subtheme 3: expectation and self-determination regarding well-being

Participants’ general expectation regarding PFME involved achieving improvement in their symptoms. Two issues were identified related to their expectations: self-efficacy expectancy and outcome expectancy. The effect of these was encouraging in terms of behavioral intentions.

*“……need to be cured…I’m able to do it."* (ID 5)

*“……expecting to stay dry…or improve."* (ID 2)

#### Subtheme 4: fear of surgery

Participants who reported positive adherence intentions mentioned the behavioral effects of fear. To avoid undesirable surgical procedures or general anesthesia, women stated that they preferred to perform the exercises regularly.

*“….if I’m not getting better… may need surgery…which I really don’t want to have.”* (ID 1)

*“….must try because I’m afraid of surgery."* (ID 4)

## Discussion

This qualitative study showed that women with SUI considered that their symptoms were possibly modifiable, that they had a negative impact on their quality of life, and that they could partially managed SUI symptoms by themselves. We also explored patients’ perceptions regarding PFME as a treatment for SUI. Overarching themes were barriers and motivators of PFME. From the perspectives of women with SUI, there is room to improve interventions in medical and nursing care for these women to eventually increase their adherence to PFME and improve their quality of life.

Pelvic floor muscle exercise is a physical therapy program used for the treatment of urinary incontinence and pelvic organ prolapse [2, 3, 26]. However, poor adherence resulting in a decline in long-term effectiveness is generally common in real-life situation [9, 10]. Reports from health care providers and women with urinary incontinence agree on the importance of “patient-related” factors in PFME adherence. The most common reasons mentioned as barriers to PFME are having trouble remembering to do the exercises and minimal perceived benefits [5, 9, 24]. Our findings among Thai women with SUI highlight two arising issues regarding PFME adherence and care process. First, fear of surgery was frequently mentioned as a strong reason to maintain a PFME routine. Some participants admitted that the fear of surgical procedures for SUI fueled their desire to perform exercises regularly so as to achieve a successful outcome. Second, some participants had difficulty identifying the pelvic floor muscle group and were unable to contract and relax their muscles correctly even after the training sessions.

Our study added valuable information from the perspectives on PFME of Asian women with SUI. Most participants specifically stated that knowledge about what causes their incontinence symptoms and how to control leakage, as well as negative experiences of those symptoms, could motivate their intention to practice the exercises, however, these were insufficient for establishing and maintaining good adherence. Self-efficacy in women’s capacity to execute regular PFME, self-determination to make choices and manage their own life, autonomy to act on their own values and interests, and identifying and preventing high-risk situations leading to poor adherence are four attributes of adherence that should be taken into account [27–29]. Regarding self-efficacy, some participants admitted that they lacked knowledge and had negative beliefs regarding PFME; thus, they considered those factors as barriers to adherence. Nevertheless, a few participants reported that they felt confident that they were doing the exercise correctly and perceived a positive effect in preventing urine leakage. Interestingly, some participants developed self-motivation out of their fear of surgery. Our findings provided thought-provoking information to enhance PFME approaches not only for SUI but also other pelvic floor symptoms. Additionally, our results provide important insight and guidance for researchers, clinic managers, and policy-makers regarding the context of barriers and motivators of PFME adherence, which would positively affect health outcomes and quality of care [6, 13].

Concerning tools, strategies and interventions to enhance long-term PFME adherence, different education techniques, mobile applications, e-health systems and vaginal devices have been studied but the results regarding adherence and incontinence symptoms are conflicting [8, 11–14, 30]. According to clinical evidences and expert opinions, identifying patient’s characteristics, barriers, and facilitators, as well as using patient-centered approaches, are crucial for improving adherence to PFME [6, 31]. Additionally, we recommend the following interventions to enable adherence on the basis of previous studies and our findings.

- Individually-based approaches should comprise knowledge and supervision. Providing clear and concise information regarding the rationale and methods of performing PFME is fundamental [18]. Then, assessing and providing real-time feedback during the exercise sessions is necessary for women to properly perform the prescribed exercises [31]. This important step seem to be neglected during routine care at our clinic. These strategies would enhance patients’ self-efficacy and self-determination and would, consequently, increase the quality of the execution and achievement of potential benefits [28].
- Group-based approaches (self-help groups and group exercise sessions), which provide mutual support and relatedness, would facilitate exercise adherence [31]. User-friendly applications or software may be used to share data (such as the number of exercises performed or incontinence symptom scores) with health care providers or patients in a group. Such approaches could increase patients’ motivation and diminish forgetfulness or competing priorities, which are high-risk factors for poor adherence [28, 31].These strategies not only increase patients’ self-determination but also serve as alternatives to routine exercise performed alone, which might bore patients and generally cause non-adherence among women.

Our study has several strengths. Primarily, we used one-on-one, in-depth interview, which provided a deep understanding of complex behaviors. This method is not only appropriate for personal and sensitive issues but also allows interviewers to probe and uncover participants’ insights. Second, the inclusion of a diverse group of providers, including a registered nurse (WS), a urogynecology nurse specialist or continence nurse (SS), and a urogynecologist (JM) on the analysis team allowed us to identify patterns and interrelationships across themes, giving a more complete picture. Additionally, reflexive exercises during the research process and participant feedback ensured the quality and robustness of our study findings [24]. Accordingly, our center will be able to apply the results of this study to improve PFME counseling strategies and increase adherence.

When drawing conclusions from the findings, it is important to acknowledge some limitations of the study. First, the authors’ professions and roles in the clinic may have affected the data collection and analysis. As a way to minimize possible impacts, all interviews were conducted by the principal investigator (WS) who did not have contact with participants in clinic activities. Second, this study included a small sample of Thai women with a very narrow age range who attended a specialized clinic in a university hospital. Thus, the findings may not be transferable to all healthcare centers or women with different age groups, ethnicities and cultural backgrounds. Nonetheless, the barriers and motivators for PFME revealed in our study are consistent with results from Western countries and those of an umbrella review regarding key factors associated with adherence to physical exercise [9, 31].

## Conclusion

The main barriers to regular PFME were lack of self-discipline, inadequate knowledge and confidence in performing the exercises, and a poor perception of PFME effectiveness. In response to the themes retrieved, proposed interventions include increasing “how to” knowledge and motivations using e-health tools and self-help groups to enhance PFME adherence, which would results in symptom reduction and quality of life improvement.

## Supplementary Information

**Additional file 1:** The interview guide.

## Data Availability

The datasets generated and/or analysed during the current study are not publicly available but are available from the corresponding author on reasonable request.

## Abbreviations

PFME: Pelvic floor muscle exercise
SUI: Stress urinary incontinence
PFMT: Pelvic floor muscle training
PFM: Pelvic floor muscle
POP–Q: Pelvic organ prolapse quantification
