# Supplementary material for: Attitudes and barriers to pelvic floor muscle exercises of women with stress urinary incontinence
Source: BMC Womens Health. 2022 Nov 26;22:477. doi: 10.1186/s12905-022-02067-4 (PMC9701389; doi:10.1186/s12905-022-02067-4)
Supplement: Supplementary file 1 — Additional file 1: The interview guide. [file 12905_2022_2067_MOESM1_ESM.docx]

**The validated open-ended questions**

1. How do you feel about your incontinence problem?
2. After receiving the information regarding pelvic floor muscle exercise, how do you feel about it?
3. You have probably heard or experienced practicing PFME. Can you tell me more about that?
4. With whom have you discussed the incontinence problem and PFME experience?
5. How do you feel about practicing PFME on your own?
6. Could you tell me what you think about PFME? What are some things you find positive? What are some things you find negative?
